# Sensitivity and Specificity of Anthropometric Indices in Identifying Obesity in Women over 40 Years of Age and Their Variability in Subsequent Decades of Life

**DOI:** 10.3390/biology11121804

**Published:** 2022-12-12

**Authors:** Anna Zwierzchowska, Joanna Kantyka, Barbara Rosołek, Agnieszka Nawrat-Szołtysik, Andrzej Małecki

**Affiliations:** 1 Institute of Sport Sciences, The Jerzy Kukuczka Academy of Physical Education in Katowice, 40065 Katowice, Poland; 2 Institute of Physiotherapy and Health Sciences, The Jerzy Kukuczka Academy of Physical Education in Katowice, 40065 Katowice, Poland

**Keywords:** anthropometric indices, obesity, women, 40+, body mass index

## Abstract

**Simple Summary:**

Anthropometric measurements and indices are a simple and inexpensive method to assess normal physical development and quickly identify the risk of diseases, including overweight and obesity. Early diagnosis of obesity supports the identification of the risk of cardiometabolic, osteoarticular, and muscular diseases, low back pain, and sarcopenia in the population. Given the changes in lifestyle and population variation, it is important to verify the diagnostic effectiveness of anthropometric measurements and indicators in different populations, taking into account groups at particular risk of developing obesity. One of them is menopausal and postmenopausal women, in whom a number of hormone-induced changes are taking place while the aging process is progressing. There is a significant change in body composition, predisposing to obesity, associated with hormonal disorders and unhealthy eating habits. The aim of our study was to find out which of the commonly used indicators for the detection of obesity is the best in the group of menopausal and postmenopausal women.

**Abstract:**

Anthropometric measurements and indices are a simple and inexpensive method to assess normal physical development and quickly identify the risk of diseases. The aim of the study was to verify the sensitivity (Se) and specificity (Sp) of selected anthropometric indices in a group of women over 40 years. The study included 87 women (group I—40 to 49 years, group II—50 to 59 years, group III—60 to 69 years, and group IV—70 to 79 years). Anthropometric characteristics were measured: body mass (BM), body height (BH), waist circumference (WC), and hip circumference (HC). Body mass index (BMI), body adiposity index (BAI), waist-hip ratio (WHR), and waist-to-height ratio (WHTR) were calculated. The percentage of fat tissue (FT) and visceral fat volume (FV) were evaluated using DEXA. A decrease in mean BH with an increase in the mean WC, WHR, and WHTR in subsequent decades. There were strong statistically significant correlations between FT and most indicators (except for WHR). FV was correlated at a strong or moderate level with most parameters. In the group of women aged 40 to 80 years, the most favorable AUC was obtained for WC, followed by BMI. BAI can be recommended as a complementary indicator to BMI.

## 1. Introduction

Anthropometric measurements and indices are a simple and inexpensive method to assess normal physical development and quickly identify the risk of diseases of affluence. They have been used in clinical and screening studies [1,2]. With their simplicity, they also allow for self-examination and self-monitoring, and thus the early diagnosis of obesity and its risks. The problem of being overweight and/or obese is increasingly affecting a large percentage of the population, especially in countries with rising economic status [3]. Obesity is also a social problem with economic consequences [4]. Therefore, education and self-monitoring that allows for early diagnosis of obesity are so important. At the same time, due to biological determinants, it is a problem that is progressive in the period of involution, that is, over 40 years of age [5], associated with an increase in the risk of cardiometabolic diseases [6], musculoskeletal diseases [7,8], and low back pain [9]. 

Early diagnosis of obesity supports the identification of the risk of cardiometabolic, osteoarticular, and muscular diseases, low back pain (LBP), and sarcopenia in population groups of different ages [10]. Anthropometric measurements and indices such as waist circumference (WC), body adiposity index (BAI), body mass index (BMI), waist-hip ratio (WHR), waist to height ratio (WHTR), which identify the risk and type of obesity, can be an antidote in the early diagnosis of obesity and are therefore an important tool for preventive measures. Despite its high diagnostic value, anthropometric measurements have some limitations in use in different population groups. For example, despite its strong correlation with body fatness, BMI does not differentiate between fat and fat-free mass [11] and also does not provide information about body fat distribution, which is important due to the comorbid metabolic diseases [12]. Furthermore, the difficult measurement of body mass in people with problems in adopting habitual posture, such as older adults with co-morbidities of the musculoskeletal system, wheelchair users, and amputees, significantly limits the possibility of using BMI in this group [13,14,15]. As an alternative to BMI, Bergman [16] proposed BAI, which is a tool that does not require weight measurement but takes into account hip circumference and body height. However, it does not provide information about body fat distribution. In this case, WC and WHR are indicators of body fat distribution. They have been shown to be of diagnostic value for identifying cardiovascular disease risks, including myocardial infarction [17] and stroke [18]. Given the changes in lifestyle and population variation, it is important to verify the diagnostic effectiveness of anthropometric measurements and indicators in different populations, taking into account groups at particular risk of developing obesity. One of them is menopausal and postmenopausal women, in whom a number of hormone-induced changes are taking place while the aging process is progressing. There is a significant change in body composition and predisposition to obesity associated with hormonal disorders and unhealthy eating habits [19]. Furthermore, bone mass density decreases, leading consequently to osteoporosis. Decreased muscle mass causes sarcopenia, which in turn can lead to frailty syndrome, thus reducing the quality of life [20]. In addition to changes due to hormonal disturbances, aging processes result in a decrease in lean body mass (including muscle mass) balanced by an increase in body fat [21]. Furthermore, after the age of 40, changes in fat distribution and the occurrence of android obesity in women are observed, which, as shown by researchers, is linked to an increase in visceral tissue [22]. The authors indicate that abdominal obesity and fatness of the internal organs are predictors in identifying risks of cardiometabolic diseases, including type 2 diabetes [23,24]. Moreover, it should be emphasized that WC is the most useful tool for identifying abdominal obesity and is already in the definition of metabolic syndrome [25].

WHO recommends indices using basic anthropometric measurements, i.e., BMI, WC, and WHR, to quickly identify the risk of cardiometabolic diseases [26]. At the same time, negative changes have been observed in lifestyle (lower physical activity, poor nutrition, stimulant use) [27]. In this context, there is a need for continuous monitoring of the sensitivity and specificity of these indicators in diverse population groups, which is the aim of our study.

Thus, population-based tools which are valid and reliable but at the same time sensitive and specific that are used in self-monitoring to support early diagnosis of obesity in menopausal and postmenopausal women are essential in counteracting its effects. Therefore, assuming that menopausal and postmenopausal women are particularly prone to overweight and obesity, an attempt was made to verify the sensitivity and specificity of selected anthropometric indices in a group of women over 40 years of age and to assess their variability in subsequent decades of life.

## 2. Materials and Methods

### 2.1. Participants

The purposeful selection was used. The study included 87 women (62.3 ± 8.3), RIDAGE Project participants. The project was aimed at the implementation of strategies supporting healthy aging. Patients participate in a range of screening tests, obtain feedback on their health and participate in lectures/workshops. Women were divided into age groups: group I—women aged 40 to 49 years (n = 6, mean age 45.5 ± 2.1 years). Group II—women aged 50 to 59 years (n = 21, mean age 54.4 ± 2.8 years), group III—women aged 60 to 69 years (n = 43, mean age 64.3 ± 3.3 years), and group IV—women aged 70 to 79 years (n = 17, mean age 73.2 ± 2.5 years).

### 2.2. Protocol of the Study

Data were collected in 2020–2022 as a part of a RIDAGE Project funded under the Ministry of Science and Higher Education’s program “Regional Excellence Initiative” in 2019–2022 (project No. 019/RID/2018/19). All measurements were carried out at the Laboratory of Body Structure, Composition, and Posture Diagnostics (RIDAGE Center, The Jerzy Kukuczka Academy of Physical Education in Katowice, Poland). Participants were informed about the advantages and disadvantages of the study and provided written informed consent. Participants were allowed to withdraw from the study at any time. The research protocol was approved by the Bioethics Committee at the Jerzy Kukuczka Academy of Physical Education in Katowice, Poland (No. 9/2012 with addendum KB/29/2020) and met the ethical standards of the Declaration of Helsinki, 2013.

### 2.3. Measurements

The direct observation method was used in the study. Measurements were made in accordance with the protocol of the certified Laboratory of Body Structure, Composition, and Posture Diagnostics at the Jerzy Kukuczka Academy of Physical Education in Katowice (PN-EN ISO 9001:2015). Each participant was examined once. The following anthropometric characteristics were measured: body mass (BM) (using a Charder MS 5410 chair scale), body height (BH) (using a Charder HM-200P stadiometer), waist circumference (WC) (using a tape measure, at the midpoint between the lower edge of the last palpable rib and the apex of the iliac crest, at the end of the expiratory phase) and hip circumference (HC) (using a tape measure, placed parallel to the ground taking into account the largest gluteal muscle circumference) [28]. BMI was calculated using the formula: BMI = BM(kg)/BH2(m), with BMI > 25 kg/m2 used as the cutoff point [29]. BAI was calculated as: (HC (cm)/BH1.5 (m)) -18, with cutoff points adopted according to Bergman [16]. WHR was calculated as WC/HC. WHTR was calculated as WC (cm)/BH (cm), with WHTR > 0.5 used as the cutoff point. For WC, the cutoff point was WC > 80 cm. The percentage of fat tissue (FT) (adopted as the gold standard) and visceral fat volume (FV) were evaluated using dual-energy X-ray absorptiometry (DEXA).

### 2.4. Statistical Analysis

Mean values and standard deviations were calculated, and the normality of the distributions (Shapiro-Wilk test) of quantitative parameters was verified. The variation in mean values of quantitative parameters between groups was verified (the non-parametric Kruskal-Wallis ANOVA on ranks test was used due to the unequal numbers in the groups). The correlations of the indicators with the gold standard (FT) and FV were calculated (Spearman correlation was used due to the small size of groups I, II, and IV). The sensitivity and specificity of the indicators were calculated taking FT as the gold standard, and the results are presented using the Receiver Operating Characteristic (ROC) curve. The AUC (Area Under ROC) was calculated. The significance level was set at *p* < 0.05.

## 3. Results

**Figure 1:**
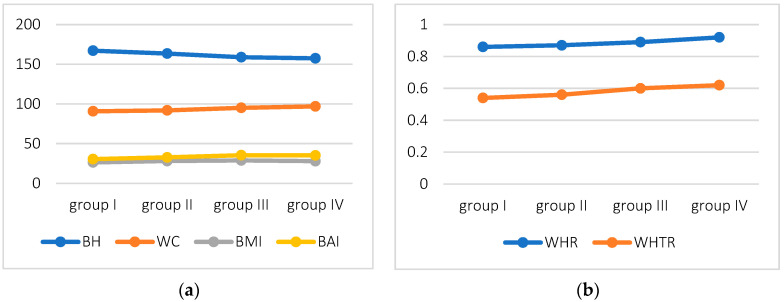
Variation of parameters in subsequent decades of life (group I—40–49 years of age, group II—50–59 years, group III—60–69 years, and group IV—70–79 years) (**a**) Variation of BH, WC, BMI, BAI; (**b**) Variation of WHR and WHTR.

The variation in BH and anthropometric indices are shown in Figure 1. A decrease in mean BH was observed, with an increase in the mean values of WC, WHR, and WHTR in subsequent decades.

Significant differences were noted for WHR (*p* < 0.038) between women in their 50s and 70s (*p* < 0.029). The mean value of body height decreased in subsequent age groups (*p* < 0.0006), and significant differences were noted between women in their 40s and 60s (*p* < 0.009), 40s and 70s (*p* < 0.003), and 50s and 70s (*p* < 0.04), see Table 1.

The correlation of FT (adopted as the gold standard) (Table 2) and FV (Table 3) with anthropometric indices (BMI, BAI, WC, WHR, and WHTR) were verified in women in the subsequent decades of life. There were strong statistically significant correlations between FT and most indicators (except for WHR).

FV was correlated at a strong or moderate level with most parameters. The exception was group I (40–49 years), with no relationship found between FV and the indicators. (see Table 3).

**Figure 2:**
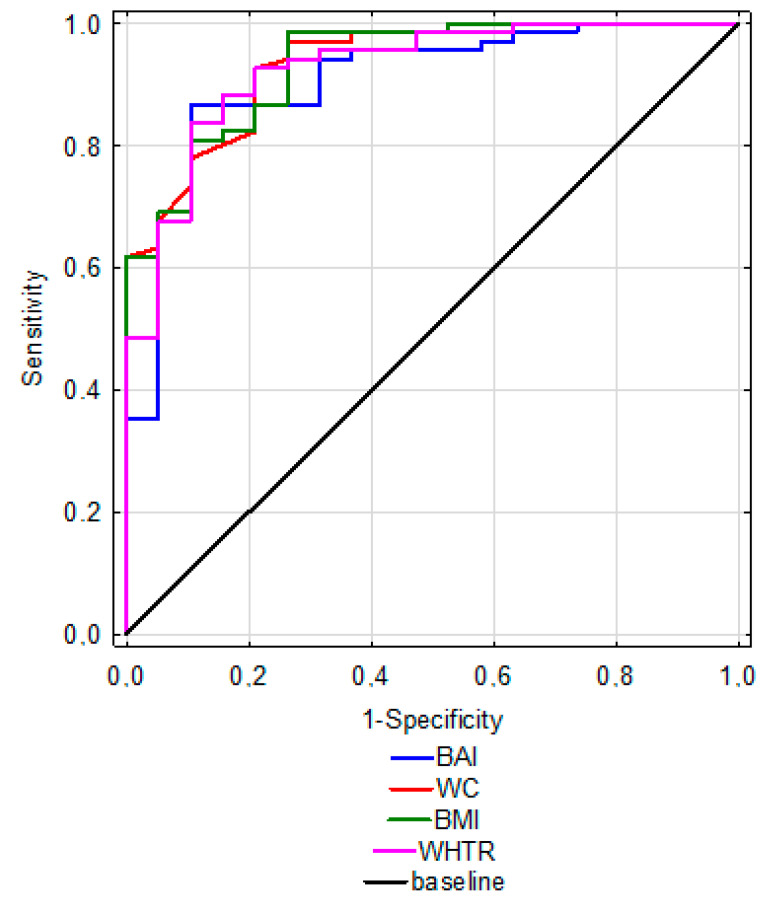
A summary of ROC curves for WC, BMI, BAI, and WHTR.

WHTR was characterized by the highest (100%) sensitivity. High sensitivity with relatively low specificity was also found for WC. The most favorable AUC was recorded for WC (0.935) and BMI (0.933) (Table 4.). Figure 2 shows a summary of ROC curves for WC, BMI, BAI, and WHTR.

## 4. Discussion

Anthropometric measurements are the primary tool in population screening to assess body composition and physique [1,2]. Due to changing lifestyles (poor nutrition, insufficient physical activity, stimulant use), there is an ontogenetic evolution in body structure and composition, which is also determined by many other factors, including hormonal [30]. Our study is consistent with such observations because WHR, which provides information about body fat distribution, was significantly differentiated over subsequent decades of life in women over 40 years of age toward android fatness. At the same time, no such variation was observed in other indicators identifying excess body weight. We only found a statistically significant variation in body height in the subsequent decades of life, as confirmed in the literature [31,32].

In our study, we also attempted to verify the sensitivity and specificity of selected anthropometric indices (BMI, BAI, WC, WHR, and WHTR) in a group of women between 40 and 80 years old, using FT obtained based on DEXA results as the gold standard. In the available literature, BMI is considered a leading and most often used screening tool for detecting overweight and/or obesity, analysis of the prevalence of overweight/obesity, and estimating their risks (e.g., cardiometabolic diseases, musculoskeletal complaints) [33,34]. We confirmed that in addition to WC, BMI is, beyond any doubt, the best classifier in identifying overweight and obesity in the group of women over 40 years of age. These findings are consistent with those reported in previous studies and allow the recommendation of these indicators as classifiers of obesity [35].

An interesting finding of our study is the 100% sensitivity of WHTR and close to 100% sensitivity (98.53%) of WC compared to the gold standard (FT). In addition to the confirmed sensitivity, both WC and WHTR correlated highly with FT and FV in subsequent decades of life. These results justify and confirm the validity of using WC in identifying the risks of metabolic syndrome, also in women over 40 years of age. Furthermore, they indicate that WHTR is effective in identifying fatness in women, which is consistent with the findings reported by Swainson [36].

However, we observed some dissonance with respect to WHR, which in the population of women aged 40–80 years, had a low statistically significant correlation(r = 0.39, *p* < 0.05) with FT (assessed by the gold standard) only in the age group of 60 to 69 years, while it correlated with FV only in the 60–69 and 70–79 groups. Caution should be exercised in interpreting this indicator on an individual basis when not dealing with excess body weight. In our view, WHR can be used complementarily with BMI, similar to BAI, which has been recommended for a decade as an alternative tool to BMI [16,37].

We showed a statistically significant correlation between BAI and FT in the women studied in all decades of life. Thus, there is no doubt that in a population of Caucasian women between 40 and 80 years old, BAI is an alternative tool for identifying fatness, which corresponds to the conclusions of the study by Elisha [38] and Djibo [30]. It is noteworthy that although the BAI fatness index showed lower correlations of FT and FV measured by DEXA (Table 3 and Table 4) than the BMI obesity index, the specificity of BAI (S = 94.74) was higher than that of BMI (S = 73.68). Such a result would support the hypothesis that in screening studies, BAI is effective in assessing a population not at risk of excessive fatness, as confirmed by the observation of Chang [39]. It is also important to recognize the importance of the BAI fatness index in terms of research protocols (the necessary measurements of somatic characteristics for calculating BAI are BH and HC) for older adults with co-morbidities of the musculoskeletal system who have difficulty adopting a habitual posture (thus making it difficult to measure body mass). Such a conclusion is consistent with findings reported by other authors [16] and those presented in our previous studies [40].

In our study, we characterized the problem of obesity/obesity indices and their sensitivity and specificity in a specific group, i.e., in menopausal and postmenopausal women. However, we also attempted to interpret these results with regard to the variation in body composition and physique in the subsequent decades of the life of women over 40 years of age, which is the strength of our research.

A weakness of our study is the small number of women in consecutive decades, which makes it impossible to verify sensitivity and specificity in each group separately, although the results of the correlations of FT and FV with selected indicators were significant (which was important for our inference). We see the possibility of extending our research with other factors determining the variability of the body structure of women in the menopausal and postmenopausal periods, e.g., taking into account the hormone replacement therapy used. We recommend this as a further research direction.

## 5. Conclusions

In the group of women aged 40 to 80 years, the most favorable AUC was obtained for WC, followed by BMI. It seems that the originality and strength of this study are that it demonstrated the high specificity of BAI and that this index can be recommended as a complementary indicator to BMI in screening studies to estimate obesity and fatness, as they can generate other pathologies associated with aging (metabolic syndrome, sarcopenia, frailty syndrome, musculoskeletal complaints). We recommend WHR only after selective identification of individuals with obesity using BMI supplemented with BAI. In our study, WHTR correlated highly with FT and, at the same time, was sensitive at 100%.

## Figures and Tables

**Table 1:** Anthropometric characteristics and indices.

Characteristics, Indices	Group I Age: 40–49 Years (n = 6)	Group II Age: 50–59 Years (n = 21)	Group III Age: 60–69 Years (n = 43)	Group IV Age: 70–79 Years (n = 17)
Age *	45.5 ± 2.1	54.4 ± 2.8	64.3 ± 3.3	73.2 ± 2.5
BH [cm] *	167.2 ± 3.9	163.5 ± 8.1	158.9 ± 5.4	157.5 ± 4.4
BM [kg]	73.6 ± 9.7	75.3 ± 15.9	72.8 ± 13.7	69.3 ± 7.7
WC [cm]	90.8 ± 12.5	91.9 ± 13.8	95.1 ± 11.5	96.9 ± 8.9
HC [cm]	104.8 ± 6.4	105.8 ± 14.1	106.7 ± 10.6	105.0 ± 7.1
BMI [kg/m^2^]	26.4 ± 4.3	28.1 ± 5.1	28.9 ± 5.3	27.9 ± 3.1
BAI [%]	30.6 ± 4.4	32.7 ± 6.6	35.4 ± 5.7	35.2 ± 4.2
WHR *	0.86 ± 0.08	0.87 ± 0.09	0.89 ± 0.06	0.92 ± 0.05
WHTR	0.54 ± 0.08	0.56 ± 0.08	0.6 ± 0.08	0.62 ± 0.06
FV [cm^3^]	1107.3 ± 382.9	1113.2 ± 740.8	1389.9 ± 716.9	1272.3 ± 583.9
FT [%]	37.3 ± 5.6	38.7 ± 7.2	40.7 ± 6.7	40.4 ± 3.9

* statistically significant variation in means between groups, BH—body height, BM—body mass, WC—waist circumference, HC—hip circumference, BMI—body mass index, BAI—body adiposity index, WHR—waist-hip ratio, WHTR—waist to height ratio, FT—the percentage of fat tissue, FV—visceral fat.

**Table 2:** Correlations of FT with indicators of obesity.

	WC	BMI	BAI	WHR	WHTR
Total					
FT [%]	0.81 ***	0.83 ***	0.76 ***	*0.19*	0.79 ***
Group I, 40–49 years (n = 7)					
FT [%]	0.89 **	0.94 **	-	0.54	0.89 *
Group II, 50–59 years (n = 21)					
FT [%]	0.91 ***	0.89 ***	0.74 ***	0.3	0.84 ***
Group III, 60–69 years (n = 43)					
FT [%]	0.83 ***	0.83 ***	0.75 ***	0.38 **	0.82 ***
Group IV, 70–79 years (n = 17)					
FT [%]	0.65 **	0.75 **	0.56 **	0.28	0.66 **

* *p* < 0.05; ** *p* < 0.01; *** *p* < 0.000; WC—waist circumference, HC—hip circumference, BMI—body mass index, BAI—body adiposity index, WHR—waist-hip ratio, WHTR—waist to height ratio, FT—the percentage of fat tissue; italics—Pearson correlation.

**Table 3:** Correlation of visceral fat volume with obesity indices.

	WC	BMI	BAI	WHR	WHTR
Total					
FV (cm^3^)	0.74 ***	0.73 ***	0.59	0.30 **	0.69 ***
Group I, 40–49 years (n = 7)					
FV (cm^3^)	−0.37	−0.25	−0.49	−0.37	−0.37
Group II, 50–59 years (n = 21)					
FV (cm^3^)	0.87 ***	0.79 ***	0.52 **	0.27	0.75 ***
Group III, 60–69 years (n = 43)					
FV (cm^3^)	0.85 ***	0.81 ***	0.69 ***	0.49 ***	0.83 ***
Group IV, 70–79 years (n = 17)					
FV (cm^3^)	0.65 ***	0.53	0.29 **	0.58 **	0.73 ***

** *p* < 0.01; *** *p* < 0.0000; WC—waist circumference, HC—hip circumference, BMI—body mass index, BAI—body adiposity index, WHR—waist-hip ratio, WHTR—waist to height ratio; FV -visceral fat, italics—Pearson correlation.

**Table 4:** Sensitivity and specificity of indicators.

	WC	BMI	BAI	WHTR
Se [%]	98.53	89.7	41.18	100
Sp [%]	57.89	73.68	94.74	84.21
AUC	0.935 *p* < 0.000	0.933 *p* < 0.000	0.906 *p* < 0.000	0.925 *p* < 0.000

Se—sensitivity, Sp—specificity, AUC—area under ROC, WC—waist circumference, HC—hip circumference, BMI—body mass index, BAI—body adiposity index, WHR—waist-hip ratio, WHTR—waist to height ratio.

## Data Availability

Access to the data can be obtained by contacting the corresponding author.
